# Not to Knot a Catheter. Case Report of the Knotting of a Suprapubic Catheter

**DOI:** 10.1100/tsw.2007.169

**Published:** 2007-06-12

**Authors:** S. A. Farook, U. Kariholu, G. Kousidis, M. Powlis

**Affiliations:** Department of Paediatric Surgery, Leeds Teaching Hospital NHS Trust, Great George Street, Leeds, LS1 3EX, UK

**Keywords:** suprapubic catheter, urethral catheter, knotting

## Abstract

A 20-month-old boy, who underwent left nephrectomy, had a suprapubic catheter inserted that knotted within the bladder. This case report identifies possible causes for such occurrences and how best to manage them.

## INTRODUCTION

Suprapubic catheters (SPCs) are commonly used to drain the bladder. The possible risks of infection and damage to the bowel have been reported in several studies. Even though the possibility of catheter knotting has been widely acknowledged, knotting of SPCs has seldom been reported.

This case report identifies the knotting of one such SPC in a child, and reviews the management and recommendations made in previous case reports.

## CASE REPORT

A 20-month-old boy was diagnosed with Wilm's tumour of the left kidney, and had to undergo left nephrectomy. Perioperatively, a 6F Cystofix SPC was inserted because the 8F urethral catheter (UC) that had been inserted did not drain well at the time of insertion. Postoperatively, both catheters were noted to be draining well. After 5 days, when attempts were made to remove the SPC, some resistance was felt, raising concerns as to whether the catheters were knotted within the bladder. Since the UC was removed with great ease, it was postulated that an isolated knotting of the SPC was a possible cause. With gentle traction, the SPC was salvaged and on close inspection, a knot was identified at the distal end (Fig. 1).

## DISCUSSION

Knotting of a catheter is a possible complication that has been widely reported, ranging from a simple UC to a Swan Ganz catheter. UC knotting is rarely seen in children under the age of 2 following the use of feeding tubes[1]; the incidence of knotting has been reported to be 0.2/100,000 catheterisations[2].

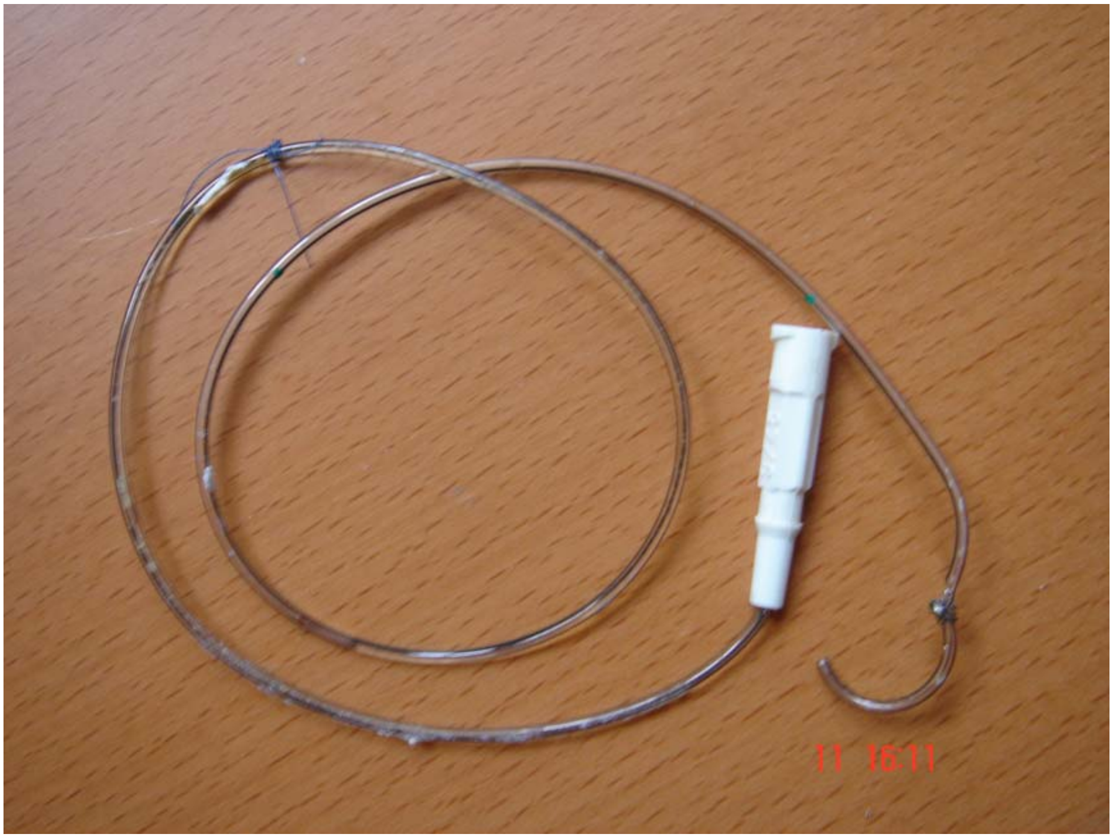

**FIGURE 1.** The SPC after removal, with the knot at the distal end.

A literature search did identify several case reports involving UCs, but very few case reports for SPC knotting. They have all highlighted a few important factors that could lead to knotting of catheters, and have made various recommendations to reduce the incidence and to minimise any further surgical intervention.

According to Arena et al., it has been hypothesised that increase in the length of the catheter within the bladder facilitates the formation of a loop, and withdrawal could lead to the knotting and tightening of the catheter[3]. Advancement of the UC could be discontinued when it starts draining the bladder, and can be secured by anchoring to adjacent tissues or inflating the balloon and facilitating the drainage of bladder contents through gravity. This is not possible with the SPC because sufficient length has to be inserted into the bladder so that residual volume is kept to a minimum. This increases the length of the catheter within the bladder and raises the possibility of knotting. Therefore, one would expect an increase in the reported cases of knotting of the SPC when compared to the UC. Whether the higher ratio of UC to SPC inserted has any association with the differences in reporting has yet to be verified.

Reviewing this case report, it is possible to postulate that the length of the catheter could have contributed to the formation of a loop or a knot, and attempts to remove it could have tightened the knot further. Although Arda et al. did highlight the possibility of bladder spasm as a contributory factor for SPC knotting, no such observation was made in this case report[4].

In case reports by Arda et al. and Gardikis et al., X-ray with contrast material was utilised to confirm the diagnosis, and the mode of removal involved a simple traction in one case report whilst the other adopted for removal via a cystotomy under general anaesthesia[4,5]. Once an attempt is made to remove the SPC and fails, one should be concerned that the catheter has knotted. It would be prudent to expose the child to unwanted radiation to confirm the likely diagnosis, but in the presence of bladder reconstruction, it would be of immense value to confirm and identify the degree of knotting, which would facilitate the selection of mode of removal. Passage of a guide wire under fluoroscopy has been used to undo the knots in UCs, which could also be considered in SPC knotting[4].

## CONCLUSION

SPC knotting is a poorly reported complication that needs to be addressed as no definite care pathway is available even in commonly reported UC knotting. After reviewing the available literature, we believe that simple steps, such as avoiding small flexible catheters, insertion of adequate length, withdrawing the catheter slowly and steadily, and even administering anticholinergic agents to reduce bladder spasms, could minimise unforeseeable complications of SPC knotting.
